# In defense of local descriptor-based few-shot object detection

**DOI:** 10.3389/fnins.2024.1349204

**Published:** 2024-02-12

**Authors:** Shichao Zhou, Haoyan Li, Zhuowei Wang, Zekai Zhang

**Affiliations:** Key Laboratory of Information and Communication Systems, Ministry of Information Industry, Beijing Information Science and Technology University, Beijing, China

**Keywords:** few-shot learning, local descriptors, contextual features, kernel method, visual similarity

## Abstract

State-of-the-art image object detection computational models require an intensive parameter fine-tuning stage (using deep convolution network, etc). with tens or hundreds of training examples. In contrast, human intelligence can robustly learn a new concept from just a few instances (i.e., few-shot detection). The distinctive perception mechanisms between these two families of systems enlighten us to revisit classical handcraft local descriptors (e.g., SIFT, HOG, etc.) as well as non-parametric visual models, which innately require no learning/training phase. Herein, we claim that the inferior performance of these local descriptors mainly results from a lack of global structure sense. To address this issue, we refine local descriptors with spatial contextual attention of neighbor affinities and then embed the local descriptors into discriminative subspace guided by Kernel-InfoNCE loss. Differing from conventional quantization of local descriptors in high-dimensional feature space or isometric dimension reduction, we actually seek a brain-inspired few-shot feature representation for the object manifold, which combines data-independent primitive representation and semantic context learning and thus helps with generalization. The obtained embeddings as pattern vectors/tensors permit us an accelerated but non-parametric visual similarity computation as the decision rule for final detection. Our approach to few-shot object detection is nearly learning-free, and experiments on remote sensing imageries (approximate 2-D affine space) confirm the efficacy of our model.

## 1 Introduction

Human intelligence can robustly learn a new concept from just a few of instances (Lake et al., 2015). For example, a child can generalize the concept of “airplane” from a single picture in a book. Yet existing supervised machine learning models need large amounts of labeled data and intensive parameters fine-tuning stage (Hinton and Salakhutdinov, 2006; Lecun et al., 2015). This motivates the setting we are interested in: “few-shot” object detection or localization, which involves searching for objects in a larger target image, given only a few query objects of these categories.

Generally, data augmentation and regularization techniques can alleviate over-fitting in low sample complexity settings for state-of-the-art image object detection computational models (e.g., deep convolution network), but do not solve it (Vinyals et al., 2016). Furthermore, a naive but much more practical approach, such as fine-tuning the model on new data, would severely over-fit. Due to the degradation on this few-shot setting, and inspired by the few-shot learning ability of humans, two recent strategies have made significant progress. One of the strategies is meta-learning, which decomposes training into an auxiliary meta-learning phase where transferable knowledge is learned, resulting in models that once trained can “learn” on new such tasks with relatively few examples (Huisman et al., 2021). The other approach is metric learning, which employs many instances of known categories to learn an embedding into a metric space where new categories are classified via proximity to the few labeled training examples embedded in the same space (Kaya and Bilge, 2019). Actually, both of the strategies still rely on large amounts of training samples. For the former, large amounts of training instances are elaborately organized into many meta-tasks, in which the training or support sets consist of several instances. For the latter, flexible combination and permutation of instances pairs/tuples demanded by the metric learning implicitly augment the training sets. Here, we claim the crucial limitation of the aforementioned methods lies in the over-parametric aspect of the utilized deep model, in which extensive training examples need to be learned by the model into its parameters.

In contrast, classical handcraft local descriptors and non-parametric models [e.g., SIFT (Lowe, 2004), and nearest neighbor classifier (Boiman et al., 2008)] allow novel examples to be rapidly assimilated while not suffering from catastrophic forgetting. Such kind of models have several intriguing advantages that are not shared by most learning-based approaches: (a) Require no training stages (i.e., lazy learning); (b) Avoid over-fitting of model parameters; (c) Can naturally handle a large number of categories via changing class/exemplars instantaneously.

Despite the aforementioned advantages, the large performance gap between traditional handcraft features, non-parametric models, and state-of-the-art deep learning-based approaches led to the perception that classical methods are not useful. Here, we claim that the capabilities of classical methods have been under-valued, especially in the few-shot setting. Specifically, the arrangements of local feature descriptors rather than themselves account for the inferior discriminative, which can be further explained as following two aspects:

1. Power law descriptor distribution gives rise to quantization errors in high-dimensional space. It is well known that densely sampled image local descriptors follow a power-law or heavy-tail distributions (Boiman et al., 2008), which imply that most descriptors would be rather isolated and found in low-density regions in the high-dimensional vector space. Furthermore, such isolated descriptors tend to be informative because they are only found in few categories but rare in other ones. In contrast, the frequent descriptors tend to appear abundantly and share among most of the classes and thus are the least discriminative for feature representation. In other words, there are almost no intuitive “clusters” in the high-dimensional space to group “visual vocabulary” with kmeans-based methods, which would consecutively degrade descriptors quantization as well as histogram scoring for global image impression.
2. Geometry preservation-based dimension reduction of descriptors makes no sense for discriminativity enhancement in the few-shot setting. It is well believed that the dimension reduction of local descriptors is essential for computational tractability and avoiding over-fitting. However, it entirely differs from the feature representation in the few-shot setting, which has not enough training instances (i.e., sparsity) to form a credible object manifold in high-dimensional feature space. In this case, the geometry preservation-based embedding of local descriptors cannot guarantee the feature discriminativity. Because the local descriptor groups only compose object instances rather than be the object instances themselves, that is, there will be no maximization interclass difference as well as separability for the embeddings in the established low-dimensional space.

To address these issues, we incorporate desirable characteristics from both parametric and non-parametric models namely, rapid acquisition of query examples while providing reliable generalization. Previous work on visual similarity in non-parametric setups has been influential on our model (Biswas and Milanfar, 2016). Herein, we propose a remarkably simple local descriptors based few-shot object detector, which requires less training costs. We focus on the context and structure information among local descriptors, which are inherently discriminative in identifying objects. Specifically, we refine local descriptors with a spatial contextual attention of neighbor affinities and then embed the local descriptors into discriminative subspace guided by Kernel-InfoNCE loss, which permits us an accelerated but non-trivial object-specific similarity computation as the decision rule for detection.

This paper is organized as follows. Section 2 briefly reports past works, which can be classified into two categories. Section 3 analyzes our motivations on the modeling of brain-inspired feature representation. Section 4 details the proposed approach. In Section 5, we compare our method with relevant few-shot object detection approaches on real-world datasets, and related analyses are also demonstrated. The conclusion is drawn in Section 6.

## 2 Related work

CNN-based representation learning methods have witnessed the improvement of object detectors (Liu et al., 2016; Redmon et al., 2016). Some of the proposed elementary tricks, such as ROI pooling (Girshick, 2015) and multi-scale feature aggregation (Lin et al., 2017), indeed adapt to few-shot settings. However, these methods generally require large amounts of training data because of their over-parametric and large-scale networks. Here, we conclude two essential paradigms related to solving the aforementioned issue: meta learning and handcraft feature representations.

### 2.1 Meta-learning

Meta-learning is a quite general learning mechanism interpreted as a “multi-task adaption process,” which mimics the capacity of human learning to learn. Given base training data (i.e., knowledge of prior tasks) and novel object categories of few supervisions to be adapted, Meta-learning devotes to a model that simultaneously detects objects from both base and novel domains.

Existing meta-learning methods are further categorized as data augmentation (Shorten and Khoshgoftaar, 2019), metric learning (Wang et al., 2019), and optimization learning (Bohdal et al., 2021). The data argumentation methods learn to generate additional examples for novel object categories to be accommodated. The metric learning methods train model to predict whether two instances belong to the same category. The optimization learning approaches specify optimization or loss functions which force faster adaptation of parameters to new categories with few examples.

Following some of the aforementioned meta-learning methods, many researchers contributed few-shot detection methods that fully exploited training data from base categories while quickly adapting the classical detection framework to predict novel classes (Finn et al., 2017), that is, most methods treat few-shot detection as an extended few-shot classification problem, ignoring the role of features for object localization. Furthermore, one can see that the data-hungry properties still exist in the meta-learning-based methods because large-scale training samples in both base class and novel ones are required, which hinders their applications in practical scenarios.

### 2.2 Hand-craft feature representations

For classical feature extraction methods, images are often represented by the collection of delicately designed local image descriptors with prior knowledge [e.g., SIFT and LARK (Seo and Milanfar, 2010)]. Specifically, these descriptors typically model the local similarity/dis-connectivity of the gray-scale, which results from the statistical facts that the image is often replete with self-similar patterns as well as abundantly appeared edges and corners.

Furthermore, the arrangement of the local descriptors also contributes to the feature discriminativity. For instance, classical “Bag of Words (BoWs)” employed normalized patches or SIFT descriptors over Difference of Gaussian, Harris-scale or Harris-affine keypoints (Mikolajczyk and Schmid, 2002), vector quantized using k-means variants. Grauman and Darrell (2005) proposed a fast kernel function that maps local descriptors to multi-resolution histograms and computes a weighted histogram intersection in feature representation space. By considering the relative position of descriptors, Biswas and Milanfar (2016) estimated a low-dimensional subspace where the original high-dimensional descriptors are embedded with their geometry intact.

## 3 Motivation

Compared with meta-learning paradigms, we endorse the handcraft feature representation methods because of their encapsulation of prior knowledge and naturally learning-free property. In addition, while contextual information is important, this issue could not be addressed by the biological-implausible BoWs, or obscured by the geometric-preserved low-dimensional embedding, which actually considers the relative position of local descriptors rather than the entire object instances, that is, the resulting scored histogram or low-dimensional embeddings cannot guarantee the desired discriminativity.

Here, we desire a brain-inspired representation learning mechanism for the challenging few-shot object detection task. It is in general acknowledged that the influence of extrinsic information on the visual representations in the brain increases with its level in the hierarchy (Kruger et al., 2013). This fact inspires us with dual principles of *reusability and composition*. Two observations argue for these motivations.

1. Reusable and less data-dependent local features. In the visual world, physical objects and scenes decompose naturally into a hierarchy of meaningful and generic parts, which could be described by local features. On the other hand, the notion of the feature itself has been already based upon the reusability assumption that similar attributes will be shared among different entities from scene to scene. These reusable local features would be sufficient to compose the large ensemble of shapes and objects that are in the repertoire of human vision (Jin and Geman, 2006). In addition, we believe that the local features are inherently data-independent because there is no report on any learning or adaptation processes in the retina and also quite some evidence on a high influence of genetic prestructuring for orientation maps in V1 (Kruger et al., 2013).
2. Semantic contexts and compositional representation learning. It is often observed that reliable object detection is notoriously difficult when purely utilizes low-level visual cues (i.e., local features) in a bottom-up inference framework, without more global contextual constraints that contribute to semantic comprehension. Actually, the semantic contexts participate in compositional representation held by humans that perceive and organize information as syntactically constrained arrangements of reusable parts. More importantly, we believe that the compositional representation should really be learned rather than hand-craft since of its semantic flexibility. This inference is supported by the neuroscience research: *learning can alter the visual feature selectivity of neurons, but the measurable changes at the single-cell level induced by learning appear to be much smaller at earlier levels in the visual hierarchy such as V1 compared to later stages such as V4 or IT* (Kruger et al., 2013). Hence, it is perhaps no coincidence that there is an apparent compositional structure in the ventral visual pathways of the more highly evolved visual systems.

## 4 Proposed method

Our brain-inspired few-shot feature representation involves object parsing, understanding, and localization from images. Specific algorithms consist of three aspects:

1. Feature representation: extract handcraft local descriptors from image patches;
2. Feature learning: learn contextual information among patches guided by Kernel-InfoNCE loss;
3. Object inference: predict object presence with cosine similarity measure.

**Figure 1:**
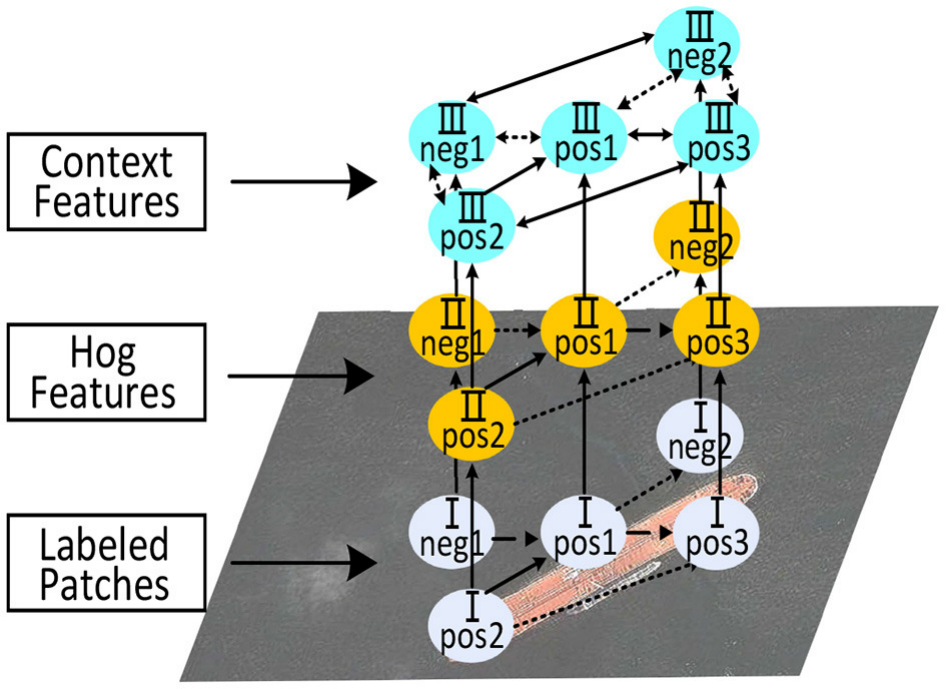
Overview of proposed model. We construct a layered model on image patches. In the bottom layer, a group of image patches is categorized as “positive” or “negative.” In the middle layer, HoG features are extracted for each patch. In the top layer, contextual relationships among these features are built with the guidance of Kernel-InfoNCE Loss.

The core of our algorithm is the first two steps as the inference step is a naive sliding window searching process. Practically, we unify feature representation and learning into a feed-forward hierarchical network that enjoys end-to-end training, as shown in Figure 1. We first introduce the proposed model (i.e., feature representation) and its training in the few-shot setting and then describe its application to the object inference.

### 4.1 Patch based representation

**Figure 2:**
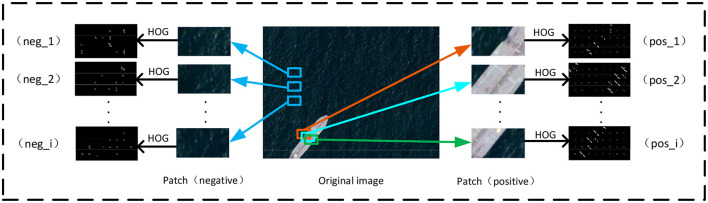
Patch-based representation. Patches are obtained from the original image. Each patch is manually labeled as “*pos*_*i*_” or “*neg*_*i*_.” HoG features are extracted for each patch.

Given only a few images (queries) containing objects of interest, we would like to know where the objects of interest lie. Note that the few-shot setting can not support modern deep neural network training without the pretraining stage. In this case, we employ patch-based image representation with hand-craft local descriptors, as shown in Figure 2. Intuitively, the dense sampling will produce many more image patches than the original large queries (i.e., implicit data augmentation), and then, a fine-grained visual parsing of the object will make sense. Moreover, the handcraft descriptors of local image patches are inherently embedded in prior knowledge, which need not be learned with amounts of training samples.

#### 4.1.1 Dense sampling and labeling

Assuming that the size of a image is *M*×*N*, we sample a dense grid of patches **X** = {**x**_1_, **x**_2_, .., **x**_*m*_} as the observations. For any image patch **x** ∈ ℝ^*p*^, we allocate a binary label **y** to indicate presence (**y** = 1) or absence (**y** = 0) of the object. The corresponding label group **Y** = {**y**_1_, **y**_2_, …**y**_*m*_} carry the information of global object presence. The relationship between **X** and **Y** is modeled by conditional probability *p*(**Y**|**X**).

With the dense sampled image patches, we can then utilize conventional intersection and concurrency ratio (IoU) to quantify whether or not the patch (partially) covers the object for following the supervised learning stage. Specifically, given pixel or bounding box-based annotations, we label a patch as positive if the IoU is greater than a threshold *t*. In this way, we can obtain a group of binary patch-based label masks as well as latent contextual information (illustrated in the next subsection) from each query image, and thus, the limited queries are fully utilized.

#### 4.1.2 Data-independent feature representation

In our current implementation, handcraft feature descriptors for representing the raw image patch were chosen to be HoG (Dalal and Triggs, 2005) without loss of generality. This type of descriptor can capture local texture information by calculating the gradient histograms (i.e., gradient direction and intensity of local regions). Actually, the essential gradient-like computations mimic the function of luminance sensitive cells with a center-surround receptive field, which emphasizes spatial change in luminance. Notably, this type of transformation into a representation emphasizing spatial change is performed at a very early stage, immediately following the receptor level, before any other visual processing takes place (Kruger et al., 2013). Hence, we advocate this data-independent and universal feature representation for the few-shot setting.

### 4.2 Context learning with Kernel-InfoNCE loss

**Figure 3:**
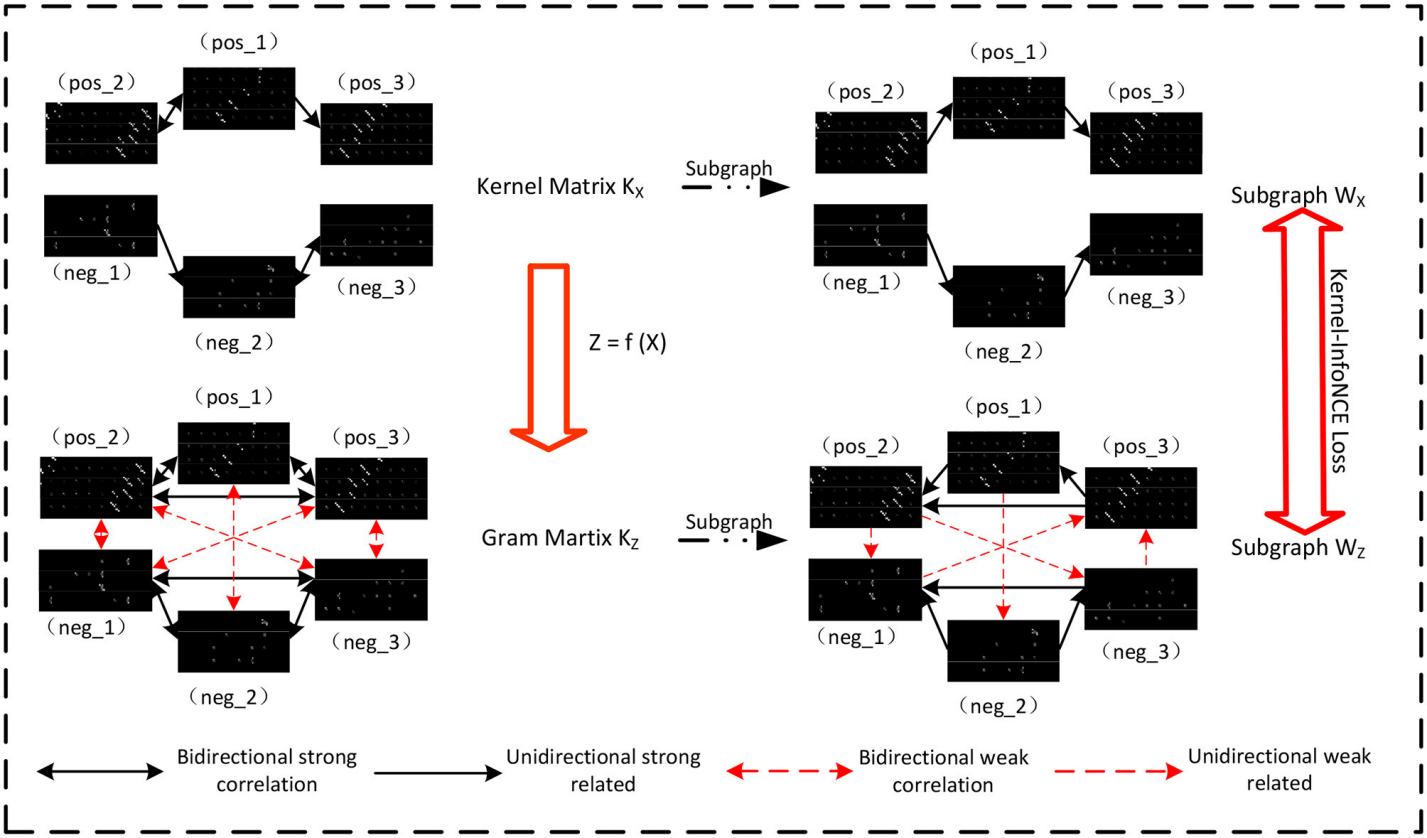
Context-aware learning. We constructed a network model, guided by the Kernel-InfoNCE loss, to learn the contextual relationships of patches.

Patch-based representation from **x**_*i*_ or their naive cascades **x** usually contain only local information about the objects, resulting in semantic ambiguities. The semantics or visual grammars are inherently discriminative cues for object detection and recognition. Thus, a further consideration of context information (i.e., the compositional structure) among patch-based representation is necessary. More importantly, while prior knowledge about image statistics points to the usefulness of gradient-like computations at the patch representation stage, there is no similar prior knowledge that would allow to design sensible transformations for the subsequent processing stage corresponding to the depths of the hierarchical visual cortex. Hence, we argue that it is one of few tractable ways of deep learning that enables the computational model to obtain the context information. Figure 3 gives the overview of the network model.

#### 4.2.1 Kernel-based context representation

Inspired by the well-established Reproducing Kernel Hilbert Space (RKHS) theory, we use kernel-based matrix to represent the contextual information between any two local descriptors within the global image. Theoretically, there is a nice duality homogeneity between inner products of (deep) feature representations and kernels. This duality can be utilized to refine neural network modules using kernels and vice-versa (Rasmussen, 2003).

Our specific implementation involves a group of *n* local descriptors **X** = {**x**_1_, **x**_2_, .., **x**_*m*_} within hand-craft feature space. For these descriptors, we can construct a kernel matrix **K**_**X**_, in which **K**_*i, j*_ = **K**(*i, j*) denotes the probability of **x**_*i*_ and **x**_*j*_ being semantically relevant defined in the initial data annotation step. Here, we adapt the most conventional translation-invariant kernel function k:X×X→ℝ, where *k*(**x**_*i*_, **x**_*j*_) is equivalent to $k^{⋆}\left({\text{x}}_{i}-{\text{x}}_{j}\right)$ for $k^{⋆}:X\to \mathbb{R}$. Classical Moore–Aronszajn's theorem states that if **K**_**X**_ is a symmetric, positive definite kernel matrix on X, there is a unique Hilbert space H on X for which *k* is a reproducing kernel. Note that it is almost impossible to calculate the semantic relevance of two descriptors using a predefined reproducing kernel in the low-level feature space because the implicitly defined feature mapping would not ideally align with the semantic meanings of specific tasks. Thus, we need to further refine the local descriptors so as to ensure the adopted translation-invariant kernel function *k*^⋆^ is still sufficient in representation, learning and inference.

Based on the established kernel, we devote to learn deep feature mapping f:X→Z. Denote **z** ≜ *f*(**x**) as the deep embedding of **x**, such that the induced Gram matrix **K**_**Z**_ representing the semantically relevant for embeddings **z** closely approximates original **K**_**X**_ as far as possible. Here, one can see that the adopted kernel representing contextual information helps guide to learn the deep feature embeddings.

#### 4.2.2 Kernel-InfoNCE loss

In this subsection, we follow the framework of kernel-based contrastive learning with Markov random fields (MRFs) (Van Assel et al., 2022; Tan et al., 2023). A whole comparison between **K**_**X**_ and **K**_**Z**_ may be difficult since there are little object samples in our few-shot setting. Consequently, we alternatively compare the MRFs of two kernels **K**_**X**_ and **K**_**Z**_. Each MRF introduces a probability distribution of unweighted directed subgraphs on the local descriptors, denoted as **W**_**X**_ and **W**_**Z**_, respectively (Van Assel et al., 2022). And then the cross-entropy loss between **W**_**X**_ and **W**_**Z**_ is naturally minimized to push the **K**_**Z**_ toward **K**_**X**_. Differently, we have artificially specified the Kernel matrix **K**_**X**_ (i.e., ground truth) for our few-shot and supervised learning scenarios.

The Kernel-InfoNCE loss, a variant of InfoNCE loss function (Chen et al., 2020), is represented as follows:


(1) $$L_{\text{Kernel-InfoNCE }}=-\log\frac{k(x,x_{i})}{\sum_{j=1}^{n}k(x,x_{j})}$$

where *k*(**x**, **x**_*i*_) denotes kernel function supported on the descriptor **x**_*i*_. Technically, the Kernel-InfoNCE loss steers low-level descriptors toward a feature space in which the positive pairs are grouped and kept away from negative ones. More importantly, the relationships between any sample pairs instead of individual instances are fully considered.

Furthermore, MRFs are employed to represent the (partial) kernel matrix in a statistical manner. Specifically, each MRF defines a probability distribution that describes an unweighted directed subgraph over kernel matrix, which is defined as Equation 2:


(2) $$S_{W}≜\{W\in {\left\{0,1\right\}}^{n\times n}|\forall (i,j)\in {\left[n\right]}^{2},W(i,i)=0\}$$

And the probability of randomly sampled subgraph (i.e., local descriptors) *P*(**W**; **K**_**X**_) is proportional to


(3) $${\text{Π}}_{(i,j)\in {\left[n\right]}^{2}}K_{X}{\left(i,j\right)}^{W(i,j)}$$

where successive multiplication in Equation 3 represents the likelihood of subgraph been sampled. In this case, the Kernel-InfoNCE loss could be refined by classical cross-entropy between **W**_**X**_ and **W**_**Z**_, which is defined as follows:


(4) $$H_{{\text{K}}_{\text{X}}}(Z)=-E_{W_{X}}[\log P(W_{Z}=W_{X};K_{Z})]$$

Benefiting to the independence of each row in the **W**_*i*_, Equation 4 could be further simplified as


(5) $$H_{{\text{K}}_{\text{X}}}(Z)=\;-\sum_{i=1}^{n}E_{W_{X}(i,\cdot )}[\log P(W_{Z}(i,\cdot )=W_{X}(i,\cdot );K_{Z})]$$

We define **P**(**W**_**Z**_(*i, j*) = 1) as the probability of node *i* pointing to node *j*, i.e., the semantic relevance between sample *i* and *j* represented by kernel matrix. Since **W**_**X**_(*i*, ·) and **W**_**Z**_(*i*, ·) can have multiple non-zero elements, this probability is no longer binary but is based on the ratio of **K**_**Z**_(*i, j*) to the sum of the weights of all outdegrees of node *i*. In this context, the cross-entropy 5 can be adjusted as follows:


(6) $$H_{{\text{K}}_{\text{X}}}(Z)=-\underset{i=1,j\neq i}{\sum^{n}}P(W_{X}(i,j)=1)\log\frac{K_{Z}(i,j)}{\sum_{k}K_{Z}(i,k)}$$

One can see that the RHS of Equation 6 is exactly the Kernel-InfoNCE loss defined in Equation 1. Technically, this formula indicates that it first samples the augmented pairs (*i, j*) for each row **i** with **P**(**W**_**X**_(*i, j*) = 1) and then optimizes the classical InfoNCE loss so as to push **K**_**Z**_ toward **K**_**X**_ with the deep feature representations f:X→Z.

### 4.3 Global inference leveraging cosine similarity measure

**Figure 4:**
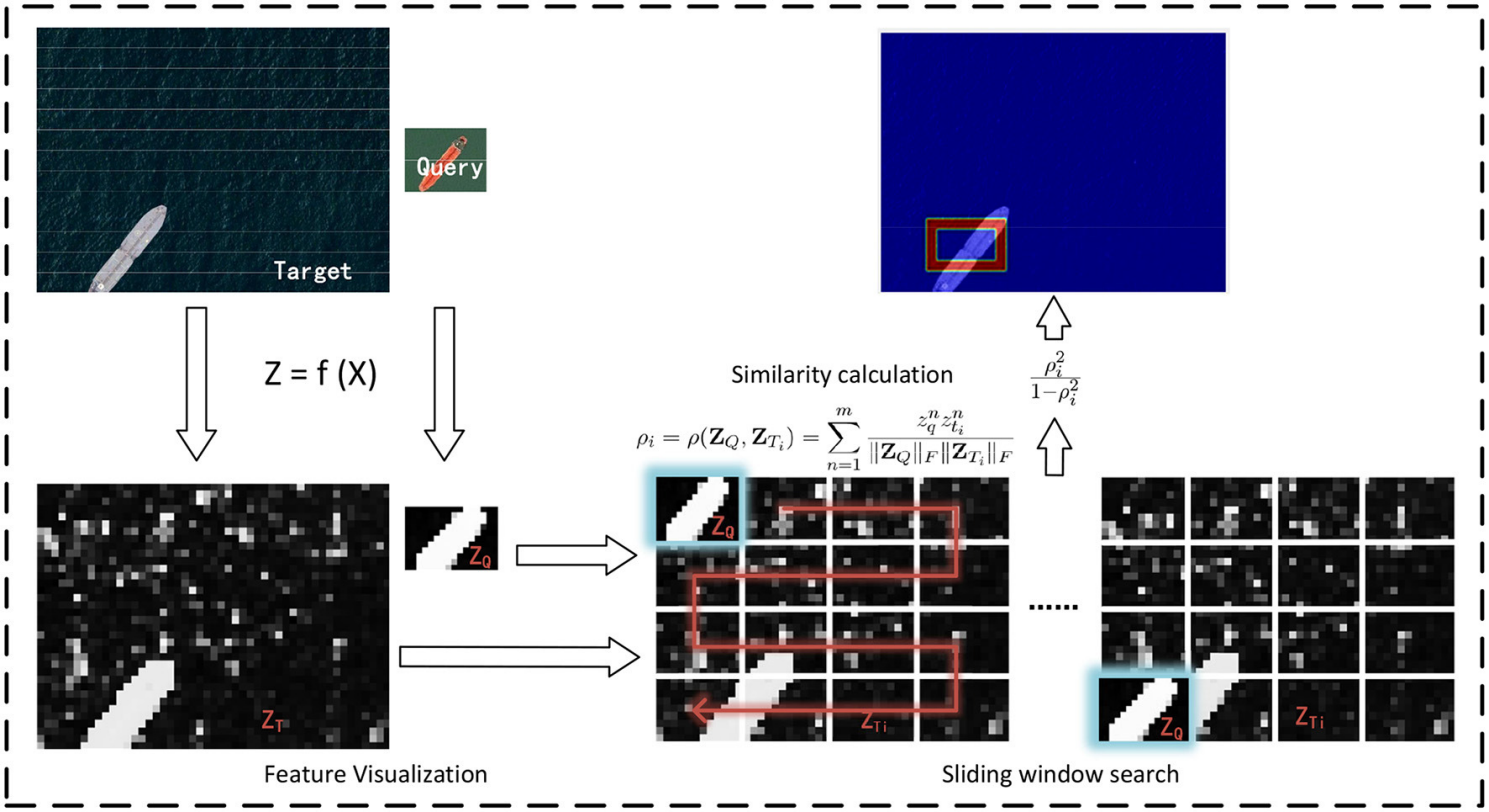
Context-aware learning. We constructed a network model, guided by the Kernel-InfoNCE loss, to learn the contextual relationships of patches.

Given the context-embedded deep features **Z** ≜ *f*(**X**) of queries, we can localize similar objects within a complete image, which is a process we refer to as global inference. To comprehensively evaluate the obtained deep features, an indiscriminate sliding window scanning is employed to predict object presence without any ROI or saliency detection preprocess. Figure 4 gives an example of global inference.

Native cosine similarity measure (i.e., inner product) is adopted to quantify the visual similarity between two deep features within each sliding window. Our specific implementation involves two vectors **Z**_*Q*_ and **Z**_*T*_*i*__, which represent the feature vectors, obtained via deep mapping *f*, of a query sample and the *i*th window, respectively. Mathematically, the visual similarity between **Z**_*Q*_ and **Z**_*T*_*i*__ is defined as follows:


(7) $$\rho (Z_{Q},Z_{T_{i}})=<Z_{Q},Z_{T_{i}}{>}_{F}=\mathrm{trace}\left(\frac{Z_{Q}^{T}Z_{T_{i}}}{\|Z_{Q}{\|}_{F}\|Z_{T_{i}}{\|}_{F}}\right)\;\epsilon [-1,1]$$

where ${\text{Z}}_{Q}=[\frac{{\text{Z}}_{Q}^{1}}{||{\text{Z}}_{Q}|{|}_{F}},...,\frac{{\text{Z}}_{Q}^{n}}{||{\text{Z}}_{Q}|{|}_{F}}]$, ${\text{Z}}_{T_{i}}=[\frac{{\text{Z}}_{T_{i}}^{1}}{||{\text{Z}}_{T_{i}}|{|}_{F}},...,\frac{{\text{Z}}_{T_{i}}^{n}}{||{\text{Z}}_{T_{i}}|{|}_{F}}]$. When we focus on each column vector *z*, Equation 7 can be rewritten as follows:


(8) $$\rho_{i}=\rho (Z_{Q},Z_{T_{i}})=\sum_{n=1}^{m}\frac{z_{q}^{n}z_{t_{i}}^{n}}{\|Z_{Q}{\|}_{F}\|Z_{T_{i}}{\|}_{F}}=\sum_{n=1}^{m}\rho (z_{q}^{n},z_{t_{i}}^{n})\frac{\left\|z_{q}^{n}\|\|z_{t_{i}}^{n}\right\|}{\|Z_{Q}{\|}_{F}\|Z_{T_{i}}{\|}_{F}}$$

Based upon the cosine similarity measure as well as sliding window scanning, we can obtain a confidence map in which the element indicates the likelihood of object presence and then place the bounding box at the high confidence region. To avoid false alarms, two simple tricks are considered in the current implementation. First, all of the likelihood value in confidence map are re-scaled with the Lawley-Hotelling Trace statistic $f\left(\rho_{i}\right)=\frac{\rho_{i}^{2}}{1-\rho_{i}^{2}}$ (Calinski et al., 2006), which suppresses the small correlation values of Equation 7 or Equation 8. Second, a conventional non-maximal value suppression process is adopted to eliminate redundant bounding boxes.

## 5 Experimental setup, results and discussions

To evaluate the effectiveness and robustness of the proposed method, we compare it with other three handcraft-based (few-shot) feature representation methods or detectors: LARK-PCA (Seo and Milanfar, 2010), LARK-LPP (Biswas and Milanfar, 2016) and sparse codes with LLE variants (hereafter called SMT) (Chen et al., 2018, 2022). Such a choice of comparison methods induces a relatively fair comparison because all of them only utilize very few queries for feature learning, rather than rely on large amounts of annotated samples (i.e., base class) for model pre-training. Notably, it is the limited queries accessibility and high utilization efficiency that accord with practical demands of few-shot learning and object detection task.

Our experiments were conducted on a high-performance server with the following configurations: Intel Gold 6330 CPU @ 2.00 GHz, NVIDIA RTX 3090 GPU, and 24GB of RAM. To fully leverage the computational power and enable efficient programming, we utilized the PyTorch framework with GPU acceleration.

### 5.1 Experimental setup

#### 5.1.1 Benchmark

We conduct the experiment on Levir dataset (Zou and Shi, 2017), from which 414 remote sensing imageries containing ocean-going ships are selected. Such choice results from a deliberated trade-off between complicated real-world scenes and synthetic ones. Technically, the utilized remote sensing imagery approximates 2-D affine plane with desired depth degradation, which is less complicated than the natural scene. Furthermore, the ocean-going ships also partly exist intractable texture clusters (e.g., trajectories and waves), which is more challenging than synthetic data. Practically, it has been mentioned in Deng et al. (2021); Han et al. (2022) that understanding of visual data collected from air platforms becomes urgently needed.

Only 21 random sampled images (i.e., objects) in the aforementioned collection were treated as queries, and the left images were designated as target images for model testing. For the queries, we randomly selected 20 ones for context learning and the left one to generate object template. In the training data setting, 4,335 small patches (32 × 32 pixels) with 10 pixels strides were sampled. Patches with an IoU above 0.3 with the target object were classified as positive samples, while those below this threshold were considered as negative ones. The reason for choosing such a small IoU value lies in the motivation that we put more emphasis on patches with salient gradient of grayscale. In addition, this setting enables the proposed model learn to keep the object away from background.

#### 5.1.2 Evaluation metrics

Precision-Recall (P-R) curve, plotting precision against recall at various confidence thresholds, is utilized to evaluate quantitative performance for each candidate few-shot detector. In addition, we highlighted equal error rate (EER), a point at which recall equals precision in the P-R curve, to indicate accuracy and reliability across each candidate detector.

#### 5.1.3 Parameter setting

To establish the deep feature embedding f:X→Z, we employ conventional ResNet-18 network (He et al., 2016) without any pretraining process, whose built-in weights were optimized with LARS optimizer. We empirically set learning rate as 1 × 10^−3^, momentum as 0.9, and weight decay as 1 × 10^−6^. The training followed an adaptive schedule across a total of 50 epochs. The inputs to the model are HoG features of patches, and the size of HoG features is 32*32.

### 5.2 Results and discussions

#### 5.2.1 P-R curve and EER evaluation

**Figure 5:**
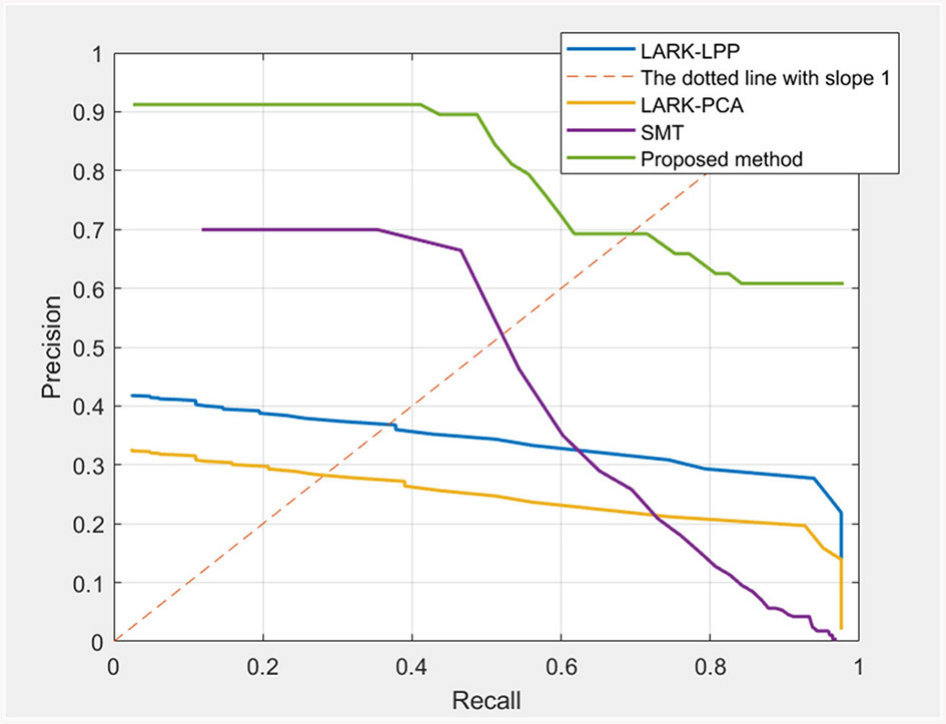
P-R curve. The values, at the intersection points of the dotted line with slope 1 and the P-R curve, represent the EER of the four evaluated methods.

Figure 5 demonstrates that the P-R curve generated by our method is better than other ones. Additionally, as shown in Table 1, our method achieves a much higher EER value of 0.692, outperforming another three ones: SMT at 0.521, LARK-LPP at 0.369, and LARK-PCA at 0.282. Such a result mainly stems from the capacity to learn deeper contextual information of the target. More importantly, all of the candidate detectors share similar local descriptors (hand-craft LARK, HOG or adaptive sparse codes), which indicates that the traditional local descriptor is actually not that “bad,” and the context information is indeed much more crucial that can not be neglected for the feature discriminativity.

**Table 1:** Comparative results of the four methods.

	**The number of queries**	**The number of targets**	**EER**
Proposed method	21	393	0.692
SMT	21	393	0.521
LARK-LPP	12	393	0.369
LARK-PCA	12	393	0.282

#### 5.2.2 Robustness to angles

**Figure 6:**
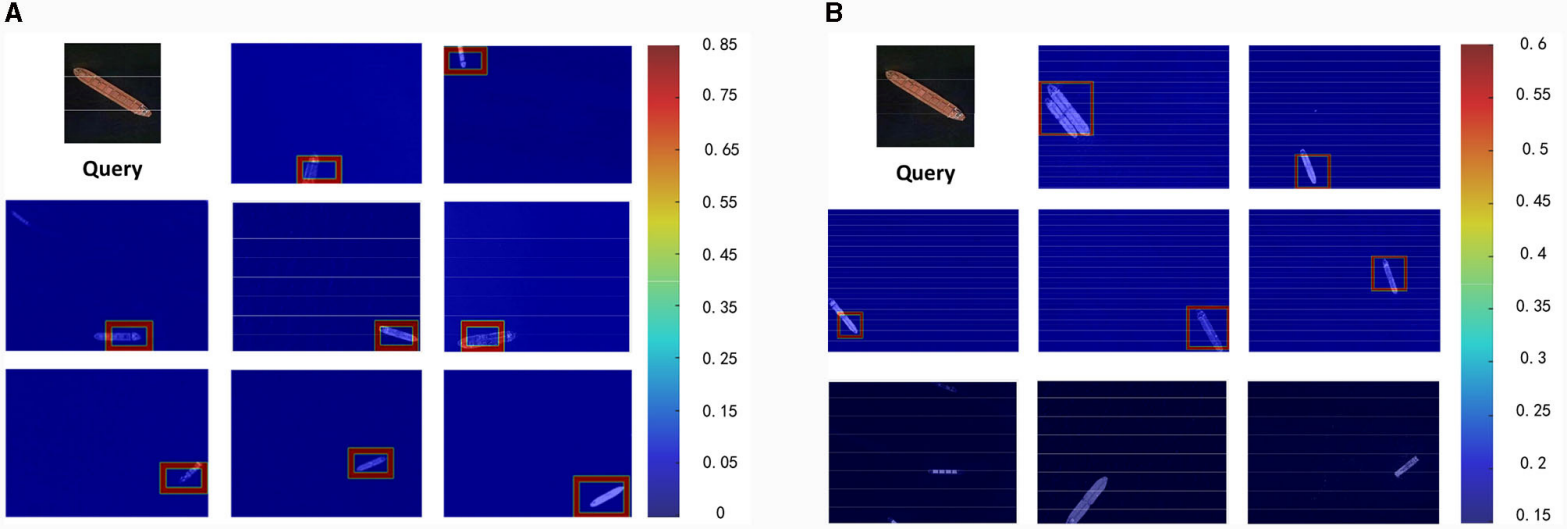
Example detections between two methods are shown here. The results from the proposed method are depicted in **(A)**. Conversely, **(B)** showcases the results by using the SMT.

Figure 6 offers a comparative analysis between our method, shown in Figure 6A, and SMT depicted in Figure 6B. Both methods utilize an equal number of query images. The comparison clearly reveals that our approach, even with a single image for generating the object template, is capable of detecting targets across a broader range of angles. On the other hand, SMT, as shown in the third line in Figure 6B, demonstrates limitations when dealing with large-angle changes. The effectiveness of our method stems from the fact that we are exploring the contextual relationships between hand-craft features, enabling the model to optimize spatial relationships and adapt more efficiently to complex angular variations.

## 6 Conclusion and future work

In this article, we have utilized classical handcraft local descriptors, which could lay foundation for learning-insensitive, effective, and efficient few-shot object detection. Given only few training samples on query targets, effectively localizing the similar regions in an imagery is a tough task given the few-shot setting that the inherently data-driven DNNs undergo. To explore such concerns, we have resorted to brain-inspired and biological-plausible computational model.

Typically, handcraft local descriptors conventionally adopted in encoding image contents have been embedded in expert visual knowledge and thus naturally have no more need of fine-tuning. However, manually arranging them without sacrificing the descriptor's discriminative power is not straightforward. To address this issue, we have studied a kernel-guided spatial context feature learning (inherently discriminative) by combining handcraft local descriptors with global semantic relevance. Our experimental results with HoG descriptor show that Kernel-InforNCE-guided context learning improves detection in comparison to PCA/LPP (with LARK descriptors) and SMT (with sparse codes) by being aware of global structure, that is, the classical local descriptor is actually not that “bad,” and the context information is much more crucial that can not be neglected for the feature discriminativity.

Our future work involves “adaptive” context learning with present kernel method “white box” deep embedding unrolling visual grammar. First, it is more reasonable that the utilized kernel matrix/graph of which meaningful edge weight assignments needs to be explicitly formulated instead of being implicitly determined in the data augmentation process. Alternatively, we will devote to exploring an explainable (deep) network layer or general module representing spatial contexts of objects. Given these improvements, other state-of-the-art few-shot detectors (e.g., meta-learning-based algorithms) will be further explored and compared in our future work.

## Data availability statement

The original contributions presented in the study are included in the article/supplementary material, further inquiries can be directed to the corresponding author.

## Author contributions

SZ: Writing—original draft. HL: Writing—review & editing. ZW: Writing—review & editing. ZZ: Writing—review & editing.
